# Characterization of patients with bacterial tracheitis after head and neck reconstruction using flaps at a high—complexity center in Bogotá, Colombia

**DOI:** 10.1016/j.jpra.2026.08.014

**Published:** 2026-08-19

**Authors:** Sebastian Alejandro Guevara-Carvajal, María Camila Rojas-Gómez, Juana Gabriela Rueda-Donado, Jacobo Moya-Beltrán, Mariana Jimenez-Fernandez, Andrés Alvarez-Tamayo, Rogers Leonardo Baquero, Martha Luz Torres-Pabon, José Alejandro Daza-Vergara, Gonzalo Mallarino-Restrepo

**Affiliations:** a Universidad del Rosario, Bogotá, Colombia; b Head & Neck Surgery Department. Hospital Universitario Mayor–Méderi, Bogotá, Colombia; c Plastic Surgery Department. Hospital Universitario Mayor–Méderi, Bogotá, Colombia; d Department of Epidemiology, Hospital Universitario Mayor, Méderi, Bogotá, Colombia

**Keywords:** Bacterial tracheitis, Tracheostomy, Head and neck cancer, Reconstructive surgery, Comorbidity

## Abstract

**Introduction:**

Tracheostomy is common in head and neck reconstructive surgeries and is associated with bacterial tracheitis. However, the influence of additional risk factors on the development of this complication remains unclear. This study aimed to estimate the incidence of bacterial tracheitis and describe associated factors in patients treated at a high-complexity center in Bogotá, Colombia.

**Methods:**

A retrospective observational cohort study was conducted at a fourth-level hospital in Bogotá between March 2023 and July 2024. Adult patients with head and neck cancer who underwent resection, flap reconstruction, and open or percutaneous tracheostomy with at least one month follow-up were included. Data were obtained from medical records, analyzed using SPSS v.31, and reported with an exploratory analysis.The study was approved by the institutional ethics committee.

**Results:**

Among 24 patients undergoing head and neck oncologic reconstruction, bacterial tracheitis occurred in 45.8% of cases. The condition was more frequent after open tracheostomy and among patients with delayed cannula replacement, although these differences were not statistically significant. *Klebsiella pneumoniae* was the predominant isolated microorganism. Patients with tracheitis had longer ICU and hospital stays, and sepsis was significantly associated with its occurrence (p = 0.031). In contrast, more frequent stoma care was associated with a lower incidence of tracheitis (p = 0.033).

**Conclusion:**

Bacterial tracheitis was a frequent complication in patients undergoing head and neck free flap reconstruction with tracheostomy. More frequent stoma care may play a protective role, although larger prospective studies are needed to better identify associated risk factors and preventive strategies.

## Introduction

In reconstructive head and neck surgery, tracheostomy is frequently performed to secure the airway, facilitate ventilation, and reduce respiratory complications in the immediate postoperative period. However, prolonged use and specific clinical conditions may predispose patients to bacterial colonization and respiratory tract infections. Bacterial tracheitis is a clinically relevant infectious complication in patients undergoing open or percutaneous tracheostomy. Its reported incidence varies across studies due to the lack of universally accepted diagnostic criteria.^1^, ^2^, ^3^

In this context, particularly in patients undergoing flap reconstruction and procedures involving manipulation of the oral cavity, oropharynx and/or airway, the prevention of infectious complications is essential to ensure favorable postoperative outcomes. A better understanding of the clinical and perioperative characteristics of patients who develop bacterial tracheitis may contribute to improving preventive strategies and optimizing clinical outcomes.^2^^,^^3^

Despite advances in surgical management and antibiotic prophylaxis protocols, evidence regarding the clinical behavior of bacterial tracheitis in adults undergoing head and neck reconstructive procedures remains limited, particularly concerning its incidence in this population. Although respiratory infections are recognized postoperative complications in head and neck surgery, bacterial tracheitis is rarely reported as a distinct clinical entity. Consequently, its epidemiology, microbiological characteristics, and clinical impact remain poorly understood.^3^ This revision better emphasizes the existing evidence regarding postoperative respiratory infections while highlighting the current knowledge gap concerning bacterial tracheitis as a distinct clinical entity.

The present study aims to estimate the incidence of bacterial tracheitis and describe the clinical and perioperative characteristics of patients who underwent open or percutaneous tracheostomy during reconstructive head and neck procedures at Hospital Universitario Mayor-Mederi (Bogotá, Colombia) between 2023 and 2024, thereby providing local evidence to support the optimization of clinical protocols and the reduction of infectious complications.

## Methods

A retrospective observational cohort study was conducted at a fourth-level hospital in Bogotá, Colombia. All patients older than 18 years with an initial diagnosis of head and neck cancer who underwent tumor resection followed by reconstructive surgery with flaps performed by the Plastic Surgery Department, and who required either open or percutaneous tracheostomy between March 2023 and July 2024, were included. Patients were required to have complete clinical information, including documentation from the first postoperative month. Exclusion criteria were a history of respiratory infections prior to surgery that could interfere with the interpretation of bacterial tracheitis, as well as patients who died during hospitalization and lacked clinical records up to the first postoperative month.

Patient identification was performed through a review of the institutional surgical registry, using the procedural code corresponding to “open and percutaneous tracheostomy” and verifying that patients had undergone a reconstructive procedure by the Plastic Surgery Department. At our institution, decisions regarding airway management are based on commonly accepted clinical criteria in head and neck oncologic surgery, including tumor location, extent of resection, anticipated airway compromise and the need for prolonged ventilatory support, as determined by the multidisciplinary surgical team. Tracheostomy is performed when these factors are expected to compromise airway patency or necessitate extended mechanical ventilation. The need for adjuvant radiotherapy is determined by the oncology team based on the definitive pathology report, which is typically available 15 to 20 days postoperatively. Since this decision is pending at the time of discharge, patients leave the hospital with the tracheostomy in place. In those who undergo radiotherapy, the tracheostomy is generally maintained for 3 to 6 months, corresponding to the duration of treatment.

Regarding the diagnosis of bacterial tracheitis, a consistent clinical approach is routinely applied. Patients with clinical suspicion undergo microbiological evaluation through tracheal secretion cultures. Clinical suspicion of bacterial tracheitis was based on findings documented in medical records, including: 1) Purulent or mucopurulent tracheal secretions visualized during suctioning or bronchoscopy, 2) Systemic signs of infection (fever > 38 °C, leukocytosis > 12,000/μL or leukopenia, 4000/μL); and 3) Absence of new or persistent pulmonary infiltrates on chest radiography obtained at the time of clinical suspicion, helping differentiate bacterial tracheitis from pneumonia. Diagnosis was confirmed by positive tracheal aspirate cultures (≥10⁵ CFU/mL). Accordingly, bacterial tracheitis was defined as the presence of compatible clinical findings together with microbiological confirmation of a pathogenic microorganism. To reduce misclassification bias, only cases with both documented clinical suspicion and microbiological confirmation were included.

In our setting, as in many resource-limited health care systems, the availability of tracheostomy devices may be restricted. As a result, non-fenestrated cuffed cannulas are frequently used initially and are subsequently replaced with fenestrated, uncuffed cannulas to facilitate speech, swallowing rehabilitation and to improve secretion clearance.

Postoperative care followed institutional clinical practice for head and neck oncologic reconstruction. Patients were routinely admitted to the intensive care unit (ICU) immediately after surgery for close monitoring of airway status, flap viability, and hemodynamic stability. After ICU discharge, patients were transferred to the inpatient ward, where multidisciplinary management was continued, including respiratory therapy, tracheostomy care, and surgical follow-up, until hospital discharge.

Data collection was carried out in REDCap and subsequently exported for statistical analysis in SPSS statistical software (version 31). A descriptive analysis was performed; categorical variables were presented as frequencies and percentages, while continuous variables were summarized using measures of central tendency and dispersion according to their distribution. The analysis aimed to characterize demographic, clinical, microbiological, and postoperative characteristics, with an exploratory comparison between patients who developed bacterial tracheitis and those who did not. Continuous variables were compared using the *t*-test or Mann–Whitney U test, as appropriate based on data distribution. Categorical variables were analyzed using the chi-square or Fisher’s exact test, depending on expected cell frequencies.

This study was approved by the research and ethics committee of Hospital Universitario Mayor-Méderi, in accordance with ethical principles for research involving human subjects and institutional standards for quality and information confidentiality.

## Results

A total of 24 patients were included, of whom 62.5% (n = 15) were men. The mean age was 57 years (range: 22–83). Most patients had a high comorbidity burden (CCI ≥3: 79.2%, n = 19). Only two patients were smokers (8.3%), one of whom developed tracheitis. Antibiotic prophylaxis prior to surgery was administered in 95.83% (n = 23) of patients, most commonly with cefazolin (75%, n = 18), followed by ampicillin/sulbactam (8.3%, n = 2).

The majority of tumors were squamous cell carcinomas (79.1%, n = 19), and most patients presented with locally advanced disease (83.3%, n = 20). Tumor histology and staging are summarized in Table 1. Regarding reconstruction, 91.7% (n = 22) underwent free flap reconstruction, while 8.3% (n = 2) underwent pedicle flap reconstruction. The most frequently used free flap was the anterolateral thigh flap (ALT) (54.2%, n = 13), followed by the fibula osteocutaneous flap (20.8%, n = 5); and the radial forearm flap in (12.5%, n = 3). Operative time was slightly longer in patients who developed tracheitis (10 h vs 9 h).

**Table 1:** Clinical characteristics in patients with vs without tracheitis.

	**With tracheitis n=11**		**Without tracheitis n=13**		**p value**
**Age**	**Mean (SD)**	**Range**	**Mean (SD)**	**Range**	0.541
	59.5 ± 17.8)	(23–83)	55.4 (± 15.1)	(22–79)	
**Sex**	**n**	**%**	**n**	**%**	0.675
Women	5	55.6%	4	44.4%	
Men	6	40%	9	60%	
**BMI**	**Mean (SD)**	**Range**	**Mean (SD)**	**Range**	0.100
	25.6 (±7.33)	(17.4–40.1)	20.8 (± 5.6)	(13.5–29.3)	
**Smoking history**	**n**	**%**	**n**	**%**	1.000
Yes	1	9.1%	1	7.69%	
No	10	90.9%	12	92.3%	
**Charlson Comorbidity Index**	**n**	**%**	**n**	**%**	1.000
2 (moderate comorbidity)	2	18.2%	3	23.1%	
≥3 (high comorbidity)	9	81.8%	10	76.9%	
**Tumor histology**	**n**	**%**	**n**	**%**	0.630
Squamous cell carcinoma (SCC)	8	72.7%	11	84.6%	
Non-Squamous cell carcinoma (SCC)*	3	27.3%	2	15.4%	
**Tumor stage**	**n**	**%**	**n**	**%**	0.717
Early stage (T1–T2, N0, M0)	9	81.8%	12	92.3%	
Locally advanced stage (T3–T4 and/or nodal involvement, M0)	1	9.1%	1	7.7%	
Metastatic stage (M1)	1	9.1%	0	0%	
**Antibiotic prophylaxis**	**n**	**%**	**n**	**%**	1.000
Yes	11	100%	12	92.3%	
No	0	0%	1	7.7%	
**Duration of antibiotic prophylaxis**	**Median (IQR)**	**Range**	**Median (IQR)**	**Range**	0.389
	1 (0–2.5)	(0–10)	1 (0–3.5)	(0–17)	
**Type of antibiotic prophylaxis**	**n**	**%**	**n**	**%**	NA
Cefazolin	8	72.7%	10	76.9%	
Ampicilin/Sulbactam	0	0%	2	15.4%	
Clindamycin	1	9.1%	0	0%	
Other regimens	2	18.2%	0	0%	
No prophylaxis	0	0%	1	7.7%	
**Tracheostomy technique**	**n**	**%**	**n**	**%**	0.123
Open	8	72.7%	5	38.5%	
Percutaneous	3	27.3%	8	61.5%	
**Type of cannula**	**n**	**%**	**n**	**%**	0.596
Fenestrated	1	9.1%	3	23.1%	
Non fenestrated	10	90.9%	10	76.9%	
**Presence of Cuff**	**n**	**%**	**n**	**%**	0.649
Cuffed	9	81.8%	9	69.2%	
Uncuffed	2	18.2%	4	30.8%	
**Type of flap reconstruction**	**n**	**%**	**n**	**%**	1.000
Free flap	10	90.9%	12	92.3%	
Pedicled flap⁎⁎	1	9.1%	1	7.7%	
**Type of free flap**	**n**	**%**	**n**	**%**	NA
Anterolateral thigh flap (ALT)	6	66.7%	7	58.3%	
Osteoseptocutaneous fibula flap	3	33.3%	3	25%	
Radial forearm flap	1	11.1%	2	16.7%	
**Operative time (hours)**	**Mean (SD)**	**Range**	**Mean (SD)**	**Range**	0.715
	10 (± 2.4)	6–13	9 (± 2.2)	6–14	
**Sepsis**	**n**	**%**	**n**	**%**	0.031*
Yes	4	36.4%	0	0%	
No	6	63.6%	13	100%	
**Septic shock**	**n**	**%**	**n**	**%**	0.458
Yes	1	9.1%	0	0%	
No	10	90.9%	13	100%	
**Hospital length of stay (days)**	**Median (IQR)**	**Range**	**Median (IQR)**	**Range**	0.052
	47 (38.5–75.5)	28–92	36 (26–44)	17–62	
**ICU length of stay (days)**	**Median (IQR)**	**Range**	**Median (IQR)**	**Range**	0.124
	17 (9.5–25)	3–32	7 (6–14)	3–21	
**Frequency of respiratory therapy**	**n**	**%**	**n**	**%**	0.682
Every <12 h	5	45.5%	8	61.6%	
Every ≥12 h	6	54.5%	5	38.5%	
**Frequency of stoma care**	**n**	**%**	**n**	**%**	0.033
Once daily	7	63.6%	2	15.4%	
Two or more times per day	4	36.4%	11	84.6%	
**Cannula replacement**	**n**	**%**	**n**	**%**	
Yes	8	72.7%	8	61.5%	0.679
No	3	27.3%	5	38.5%	
**Frequency of cannula replacement**	**n**	**%**	**n**	**%**	0.315
<30 days	3	37.5%	6	75%	
30 days or more	5	62.5%	2	25%	
**Reintervention due to complications**	**n**	**%**	**n**	**%**	0.414
Yes	7	63.6%	5	38.5%	
No	4	36.4%	8	61.5%	
**In hospital-mortality**	**n**	**%**	**n**	**%**	1.000
Yes	1	9.1%	2	15.4%	
No	10	90.9%	11	84.6%	

NA: Not Applicable.

Continuous variables were compared using the *t*-test or Mann–Whitney U test depending on distribution. Categorical variables were compared using the chi-square or Fisher’s exact test.

⁎ The non-SCC group included adenoid cystic carcinoma (n = 2), neuroblastoma (n = 1), soft tissue sarcoma (n = 1), and papillary thyroid carcinoma (n = 1).

⁎⁎ The two pedicled flaps corresponded to a pectoralis major flap and a supraclavicular flap.

Concerning tracheostomy technique, 54.2% (n = 13) of patients were treated with an open approach, while 45.8% (n = 11) underwent a percutaneous approach. Overall, 11 patients (45.8%) developed bacterial tracheitis. The proportion of tracheitis was higher among patients who underwent open tracheostomy compared with those who underwent a percutaneous approach (61.5%vs. 27.3%); however, this difference did not reach statistical significance (p = 0.123). No significant differences were observed in sex distribution between patients with and without tracheitis (p = 0.675). Detailed comparisons are shown in Table 1.

A total of 16 microorganisms were isolated from 11 patients with bacterial tracheitis, of whom four (36.3%) had polymicrobial infections. The most commonly identified microorganism was *Klebsiella pneumoniae* (37.5%). *Proteus mirabilis, Escherichia coli* and *Enterobacter hormaechei* each accounted for 12.5% of isolates. Less frequently identified microorganisms (6.3% each) included *Pseudomonas aeruginosa, Streptococcus pneumoniae, Acinetobacter baumannii* and *Stenotrophomonas maltophilia*. Microbiological findings are summarized in Table 2.

**Table 2:** Microorganisms isolated in patients with bacterial tracheitis*.

	**n**	**%**
*Klebsiella pneumoniae*	6	37.5%
*Proteus mirabilis*	2	12.5%
*E. coli*	2	12.5%
*Enterobacter* hormaechei	2	12.5%
*Pseudomonas aeruginosa*	1	6.3%
*Streptococcus pneumoniae*	1	6.3%
Acinetobacter baumanii	1	6.3%
Stenotrophomona maltophilia	1	6.3%

⁎ A total of 16 bacteria were isolated among the 11 patients with tracheitis. Four patients had polymicrobial infections.

Patients who developed tracheitis had a longer median ICU length of stay compared to those who did not (17 days [IQR: 9.5–25] vs. 7 days [IQR: 6–14]), although this difference was not statistically significant (p = 0.124). Similarly, hospital length of stay was longer in patients with tracheitis (47 days [IQR: 38.5–75.5] vs. 36 days [IQR: 26–44]), without reaching statistical significance (p = 0.052).

Regarding postoperative care, cannula replacement was primarily performed to facilitate rehabilitation (n = 14), while two replacements were performed due to device-related complications (one due to cuff dysfunction and one due to accidental decannulation). When stratified by timing of cannula replacement, early replacement (<30 days) was more frequent among patients without tracheitis (75%vs. 37.5%), whereas delayed replacement (≥30 days) was more common in patients who developed tracheitis (62.5%vs. 25%). Despite this observed trend, the difference did not reach statistical significance (p = 0.315).

A significant difference was observed in the frequency of stoma care between groups (p = 0.033). Patients who developed tracheitis were more likely to receive less frequent stoma care (63.6%, n = 7, vs. 15.4%, n = 2), while more frequent care (two or more times per day) was more common in patients without tracheitis (84.6%, n = 11 vs. 36.4%, n = 4).

Respiratory therapy was administered to all patients and included airway tracheal suctioning, and bronchial hygiene maneuvers. No significant differences were observed in its frequency between groups. Patients receiving therapy every < 12 h accounted for 45.5% (n = 5) of those with tracheitis and 61.6% (n = 8) of those without, while therapy every ≥ 12 h was observed in 54.5% (n = 6) and 38.5% (n = 5), respectively (p = 0.682).

Over the course of hospitalization, four patients with tracheitis presented sepsis, representing a significant association between sepsis and tracheitis (p = 0.031). Among these patients, microbiological concordance between tracheal aspirate and blood cultures was observed in three cases, supporting the respiratory tract as the probable source of bloodstream infection. In the remaining patient, blood cultures remained negative after 72 h of incubation. These findings suggest that bacterial tracheitis may contribute to systemic infectious complications in susceptible patients undergoing major head and neck reconstruction.

Three deaths were recorded (12.5%, n = 3). One death was due to sepsis, and two were related to oncologic progression. One case involved a patient with olfactory neuroblastoma who presented early progression at 30 days with intracranial involvement and nodal metastases, complicated by cardiorespiratory arrest. The other was a patient with tongue squamous cell carcinoma and positive margins who underwent euthanasia at 39 days postoperatively.

Reintervention defined as an unplanned return to the operating room due to complications within the first 24 h after surgery, was more frequent in patients with tracheitis (63.6%, n = 7 vs. 38.5%, n = 5), although this difference was not statistically significant (p = 0.414). Among patients with tracheitis, abscess was the most frequent indication for reintervention (28.5%), followed by hematoma, bone exposure, and flap-related complications, each occurring in 14.3% of cases. The distribution of reinterventions is shown in Table 3.

**Table 3:** Causes of reintervention in patients with vs without tracheitis.

	**With tracheitisn=7**		**Without tracheitis n=5**	
	**n**	**%**	**n**	**%**
Hematoma	1	14.3%	0	0%
Abscess	2	28.5%	1	20%
Bone exposure	1	14.3%	0	0%
Oronasal fistula	0	0%	1	20%
Revision of venous anastomosis due to thrombosis	1	14.3%	1	20%
Partial flap necrosis	1	14.3%	1	20%
Total flap loss	1	14.3%	1	20%

## Discussion

Postsurgical tracheitis in patients who undergo flap reconstructions plays an important role due to its impact on morbidity and surgical outcomes. The observed incidence of bacterial tracheitis in this study was 45.8%, which is consistent with the literature. Wijayarathna et al. reported 32% of respirator infections in oral cavity surgery with free flap reconstruction, with 36% occurring in tracheostomized patients.^4^ Similarly, Pecorari et al. documented respiratory infections as the most common postoperative complication in head and neck cancer surgery, with tracheostomy identified as a significant risk factor in multivariate analysis.^1^

In our study, bacterial tracheitis was more frequently observed in patients with CCI ≥3 (81.8%, n = 9), although this association did not reach statistical significance. Similar findings have been reported by Glugliotta et al., who demonstrated a higher frequency of postoperative infectious complications among patients with higher ASA classifications (p = 0.01).^6^ Considering that most patients in our study had high comorbidity burden, these results support the potential role of baseline medical status in increasing susceptibility to postoperative infections. In line with this, a recent meta-analysis identified comorbidity burden as an independent risk factor for surgical site infections in head and neck surgery.^7^ Therefore, the CCI may represent a useful tool for identifying patients at higher infectious risk and guiding preventive postoperative care strategies.

As part of standard perioperative care at our institution, all patients undergoing major head and neck surgery with microvascular reconstruction receive antibiotic prophylaxis. Several studies have compared the efficacy of cephalosporins over amoxicillin/clavulanate in tracheitis-related studies, with no significant differences observed.^8^^,^^9^ Some authors have suggested that the addition of metronidazole to cefazolin could reduce the risk of infection by broadening anaerobic coverage, although the evidence regarding this benefit is inconclusive.^10^, ^11^, ^12^ A recent meta-analysis suggested that cefazolin, ampicillin/sulbactam, and amoxicillin/clavulanate are the preferred antibiotic for preventing surgical-related infections, whereas clindamycin is associated with a higher risk of surgical site infection (OR = 2.73; 95% CI: 1.50–4.97; p = 0001) and should not be used as the only antibiotic in this setting. Furthermore, it has been shown that extending prophylaxis beyond 48 h does not provide additional benefit in reducing infectious complications, with a duration of 24–48 h being sufficient even in higher-risk patients.^13^ In our study, most patients received cefazolin as prophylaxis; however, no association was found between the choice of prophylactic antibiotic and the subsequent development of bacterial tracheitis.

It is important to note that perioperative antibiotic prophylaxis is intended to prevent surgical site infections, not respiratory tract infections such as bacterial tracheitis. The organisms identified in tracheal cultures from patients who developed tracheitis, including multidrug resistant pathogens reflect healthcare-associated colonization and infection occurring days to weeks postoperatively, rather than perioperative contamination.^14^ The presence of resistant organisms in postoperatively respiratory cultures does not justify the routine use of broader-spectrum prophylactic antibiotics, as this approach lacks evidence for preventing tracheitis and may contribute to further antimicrobial resistance.

The main microorganisms isolated in tracheitis cases in our population were, in order of frequency: *Klebsiella pneumoniae (37.5%), Proteus mirabilis, E. coli, Enterobacter hormaechei, and Stenotrophomonas maltophilia* (each 12.5%), followed by *Pseudomonas aeruginosa, Streptococcus pneumoniae*, and *Acinetobacter baumannii* (each 6.3%). These results are consistent with those reported in other studies, such as the one conducted by Blot et al., in which several microorganisms were isolated from adult patients undergoing airway manipulation through mechanical ventilation or tracheostomy, including *Pseudomonas aeruginosa, Acinetobacter baumannii, Klebsiella* spp.*, E. coli, Staphylococcus aureus, Streptococcus pneumoniae, Enterococcus* spp.*, and Bacillus cereus*.^15^ Similarly, Quintero et al., in a study conducted at our institution, found that *Klebsiella pneumoniae* and *Acinetobacter baumannii* topped the frequency list in postsurgical tracheitis cultures.^16^ The clinical implications of these findings are significant. Cefazolin, which was used in 75% of our patients as prophylaxis, has limited coverage against gram-negative organisms producing beta-lactamases, which may partially explain the high incidence of tracheitis observed. These findings suggest that broader-spectrum prophylactic strategies may need to be considered in this population.

Tracheostomy technique may also be associated with postoperative outcomes. A systematic review by Brass et al. demonstrated that the percutaneous technique reduces the risk of wound infection/stomatitis by 76% compared with the open technique (RR 0.24, 95% Cl: 0.15–0.37, p < 0.00,001).^17^ The proposed mechanism includes reduced tissue manipulation and less tissue trauma with the percutaneous approach.^18^ A previous study conducted at our same institution, in a different patient population, reported postoperative tracheitis in 32.5% of patients who underwent open tracheostomy and 25% of those who underwent a percutaneous approach, with no statistically significant difference between techniques (p = 0.287).^16^ Similarly, in our study, tracheitis was more frequently observed in patients who underwent the open technique (72.7%, n = 8) compared with those who underwent a percutaneous approach (27.3%, n = 3); however, this difference did not reach statistical significance.

In our study, longer operative time (≥9 h) was more frequently observed among patients who developed bacterial tracheitis, possibly reflecting greater surgical complexity and prolonged exposure to potential sources of infection; however, this difference did not reach statistical significance. Prolonged operative time has been consistently identified as a risk factor for postoperative infections. For instance, Pecorari et al. reported longer surgical duration as an independent risk factor in multivariate analysis for postoperative infections.^1^ Similarly, Wang et al., in a meta-analysis, confirmed operative time as a significant predictor of surgical site infection (OR 1.42, 95% Cl: 1.16–1.74).^7^

Concerning tracheostomy management, standard postoperative practice at our institution involves replacing a non-fenestrated cuffed tube with a fenestrated non-cuffed tube to facilitate patient rehabilitation (promote phonation and improve secretion clearance). In our study, bacterial tracheitis was diagnosed at a mean of 11.4 ± 6.5 days after surgery, ranging from postoperative day 4 to day 26. Patients in whom this change was delayed beyond 30 days showed a higher proportion of tracheitis compared with those who underwent earlier replacement; however, this difference did not reach statistical significance, likely due to limited sample size. Given the retrospective nature of this study and the timing of tracheostomy placement during the index surgery, we acknowledge that the observed associated between delayed cannula replacement and tracheitis may be bidirectional. While delayed replacement could theoretically increase infection risk through prolonged exposure to cuffed tubes, it is also possible that early development of tracheitis may have influenced clinical decisions to delay cannula changes. The design of our analysis limits our ability to establish temporal causality in this relationship.

Nevertheless, there is evidence supporting the potential role of tube type and replacement timing as contributing factors to infection risk. In a cross-sectional study of tracheostomized patients undergoing cannula replacement, Yanti et al. identified a replacement interval longer than 30 days as an independent risk factor for tracheostomy stoma infection (OR 10.66, 95% Cl: 1.07–105.99, p = 0.043).^19^ In settings where both tube types are available and invasive mechanical ventilation is not anticipated, the immediate use of uncuffed tracheostomy tubes may be considered. Patel et al. reported that this approach was associated with shorter hospital length of stay (7.7 vs. 9.7 days, p < 0.001) without an increase in respiratory complications in patients undergoing head and neck free flap reconstruction.^20^ This is further supported by Kumarasinghe et al., who demonstrated that cuffed tracheostomy tubes are associated with significantly higher rates of microbial colonization compared to uncuffed tubes (66.7%vs. 39.1%, p = 0.032).^5^ The proposed mechanism includes the formation of folds in the inflated cuff that allow microaspiration of oropharyngeal secretions, as well as biofilm formation on the internal surface of the tube, which sustains bacterial colonization of the lower respiratory tract.^21^ These findings reinforce the rationale for early transition to uncuffed devices when clinically feasible.

Higher frequency in stoma wound care was associated with a lower incidence of tracheitis (p = 0.033), representing one of the most significant and clinically actionable findings of this study. This is consistent with the previously mentioned study, in which wound care performed only once per day was associated with up to a 9.3-fold increased risk of infection (OR 9.36, 95% CI: 1.53–57.09, p = 0.015), highlighting the importance of adequate hygiene as part of baseline care.^18^

Regarding hospital stay, we found that patients with tracheitis had longer total hospitalization (47 vs. 36 days) and longer ICU stays (17 vs. 7 days), although neither difference reached statistical significance. These findings are consistent with those reported by Glugliotta et al., who documented that both ICU and total hospitalization time are associated with a higher incidence of infectious complications (p = <0,05).^6^ Notably, this relationship is likely bidirectional: prolonged hospitalization increases exposure to nosocomial pathogens, while infectious complications may themselves delay recovery and extend hospital stay. Therefore, the longer hospitalization observed in patients with tracheitis cannot be attributed solely to the infection and may also reflect overall clinical complexity. Reinterventions were more frequent in patients with tracheitis (63.6%vs. 38.5%, p = 0.414), although this difference was not statistically significant and the indications for reoperation were heterogeneous, precluding a direct causal link with tracheitis.

This study presents several limitations that should be considered. First, the small sample size and the descriptive study design do not allow causal relationships to be established. Second, the retrospective review of medical records carries an inherent risk of information bias, as the analysis depends on the accuracy and completeness of the clinical documentation. Some variables may have been incomplete recorded, which could lead to misclassification or missing data. In addition, because microbiological cultures were not performed systematically, the true incidence of bacterial tracheitis may have been underestimated or overestimated. Despite these limitations, this study provides relevant information about our local population, for which previous reports are limited, and contributes data from a geographical setting that is underrepresented in the international literature. These findings may serve as a basis for future studies with larger samples and prospective designs.

## Conclusions

Bacterial tracheitis was observed in 45.8% of patients undergoing head and neck free flap reconstruction with tracheostomy. Stoma care frequency and the occurrence of sepsis were the only variables that reached statistical significance in their association with tracheitis. The remaining clinical and surgical variables analyzed did not show statistically significant differences between groups. These findings, derived from a small retrospective study, should be considered preliminary and may serve as a basis for future prospective studies in this population.

## Informed consent and patient data

Written informed consent was obtained from all participants prior to inclusion in the study. All data was anonymized to ensure confidentiality.

## Funding

No external funding was received.

## Ethical approval

Approved by the institutional review board.

## Generative artificial intelligence

During the preparation of this work, the author(s) used ChatGPT solely for grammar and punctuation editing. The authors reviewed and edited the content as needed, and they take full responsibility for the content of the published article.

## CRediT authorship contribution statement

**Sebastian Alejandro Guevara-Carvajal:** Investigation, Data curation, Writing – original draft. **María Camila Rojas-Gómez:** Investigation, Data curation, Formal analysis, Writing – original draft. **Juana Gabriela Rueda-Donado:** Investigation, Data curation, Writing – original draft. **Jacobo Moya-Beltrán:** Investigation, Data curation, Writing – original draft. **Mariana Jimenez-Fernandez:** Investigation, Data curation, Writing – original draft. **Andrés Alvarez-Tamayo:** Writing – review & editing. **Rogers Leonardo Baquero:** Writing – review & editing. **Martha Luz Torres-Pabon:** Writing – review & editing. **José Alejandro Daza-Vergara:** Formal analysis. **Gonzalo Mallarino-Restrepo:** Supervision, Writing – review & editing, Project administration.

## Declaration of competing interest

The authors declare no conflicts of interest.

## Data Availability

The data supporting this study are available from the corresponding author upon reasonable request.
